# Visceral larva Migrans in a Young Italian Patient: A Diagnostic Dilemma

**DOI:** 10.1007/s11686-023-00723-9

**Published:** 2023-10-23

**Authors:** Emanuela Francalanci, Tommaso Manciulli, Giulia Bandini, Pierluigi Blanc, Sara Irene Bonelli, Enrico Brunetti, Eduardo Gotuzzo, Carmen Michaela Cretu, Federico Gobbi, Alessandro Bartoloni, Lorenzo Zammarchi

**Affiliations:** 1 Department of Clinical and Experimental Medicine, University of Florence, Florence, Italy; 2 Department of Internal Medicine, Careggi University Hospital, Florence, Italy; 3 Unit of Infectious Diseases, Pistoia Public Hospital, Azienda USL Toscana Centro, Pistoia, Italy; 4 Department of Clinical-Surgical, Diagnostic and Pediatric Sciences, University of Pavia, Pavia, Italy; 5 Instituto de Medicina Tropical Alexander Von Humboldt, University Cayetano Heredia, Lima, Peru; 6 Department of Parasitology, Carol Davila University of Medicine and Pharmacy, Colentina Clinical Hospital, Bucharest, Romania; 7 Department of Infectious/Tropical Diseases and Microbiology, IRCCS Sacro Cuore-Don Calabria Hospital, Negrar, Verona, Italy; 8 WHO-Collaborating Center for the Clinical Management of Cystic Echinoccocosis, San Matteo Hospital Foundation, Pavia, 27100 Italy; 9 Regional Referral Center for Tropical Diseases, Careggi University Hospital, Florence, Italy; 10 Infectious and Tropical Diseases Unit, Careggi University Hospital, Florence, Italy

**Keywords:** Visceral larva migrans, Toxocariasis, Fascioliasis, Focal liver lesion, Diagnosis, Serology

## Abstract

**Background:**

The association of fever, focal hepatic lesions and peripheral hyper-eosinophilia (FHLH) can be observed in both infectious and non-infectious conditions. Fascioliasis, capillariasis, toxocariasis, all causes of visceral larva migrans (VLM), represent most of the former, whilst lymphomas, eosinophilic leukemias and mastocytosis belong in the non-infectious conditions.

**Methods:**

We prospectively followed a young patient presenting with FHLH in the Tuscany region of Italy.

**Results:**

The patient was subject to serological and parasitological examination in an attempt to clarify the origin of the lesions. Serologies for both *Fasciola hepatica* and *Toxocara* spp. were positive, with the latter presenting a higher index. We opted for treatment with a prolonged course of albendazole due to the serological results and being toxocariasis more frequent in our setting. The patient was then subject to radiological follow-up. The patient responded to treatment with albendazole as shown by a decrease in eosinophils, seronegativization for *Toxocara* spp., clinical and radiological improvement. Toxocariasis was hence considered the most likely diagnosis.

**Conclusions:**

Parasitic infections cannot be disregarded in the presence of FHLH. Differential diagnosis between these parasitic infections can be challenging due to the presence of similar clinical presentations and serological cross-reactions, and follow-up of the patient is needed to ensure optimal treatment outcomes.

**Supplementary Information:**

The online version contains supplementary material available at 10.1007/s11686-023-00723-9.

## Background

Visceral larva migrans (VLM) is a syndrome caused by the migration of helminths through the liver or other organs [15]. It is a protean disease, with clinical manifestations ranging from skin disorders (pruritus, rash, urticaria and angioedema) to lung involvement (asthma-like symptoms and pleural effusion) and diffuse abdominal involvement (abdominal effusion, hepatosplenomegaly). Focal hepatic lesions and peripheral hypereosinophilia (FHLH) or ocular involvement (ocular larva migrans) may be also frequent. Manifestations depend on the host immunological status and the parasitic burden [10, 19]. It is amongst the causes of focal liver lesions with associated eosinophilia that can pose a diagnostic challenge to clinicians. Non-infectious causes include malignant lesions such as lymphomas or liver localizations of eosinophilic leukemias [8, 17]. Autoimmune diseases such as systemic mastocytosis can also localise in the liver [6].

Nematodes belonging to the genus *Toxocara* (mainly due to *Toxocara canis* or *Toxocara cati*, less frequently due to, *Toxocara vitulorum*, *Toxocara pteropodis*) are the main causes of VLM [2, 5]. Toxocariasis is a cosmopolitan infection [10] which can cause ocular and neurological manifestations, such as meningitis, encephalitis, myelitis, cerebral vasculitis [13]. Other known causes of VLM include other parasites, such as *Ascaris suum*, *Gnathostoma spp* [16], *Baylisascaris spp* [9] and capillariasis [12]. We report the case of a young patient with a probable diagnosis of liver toxocariasis seen in our outpatient department after referral from a first-line hospital in the Tuscany region of Italy.

## Case Presentation

**Fig. 1:**
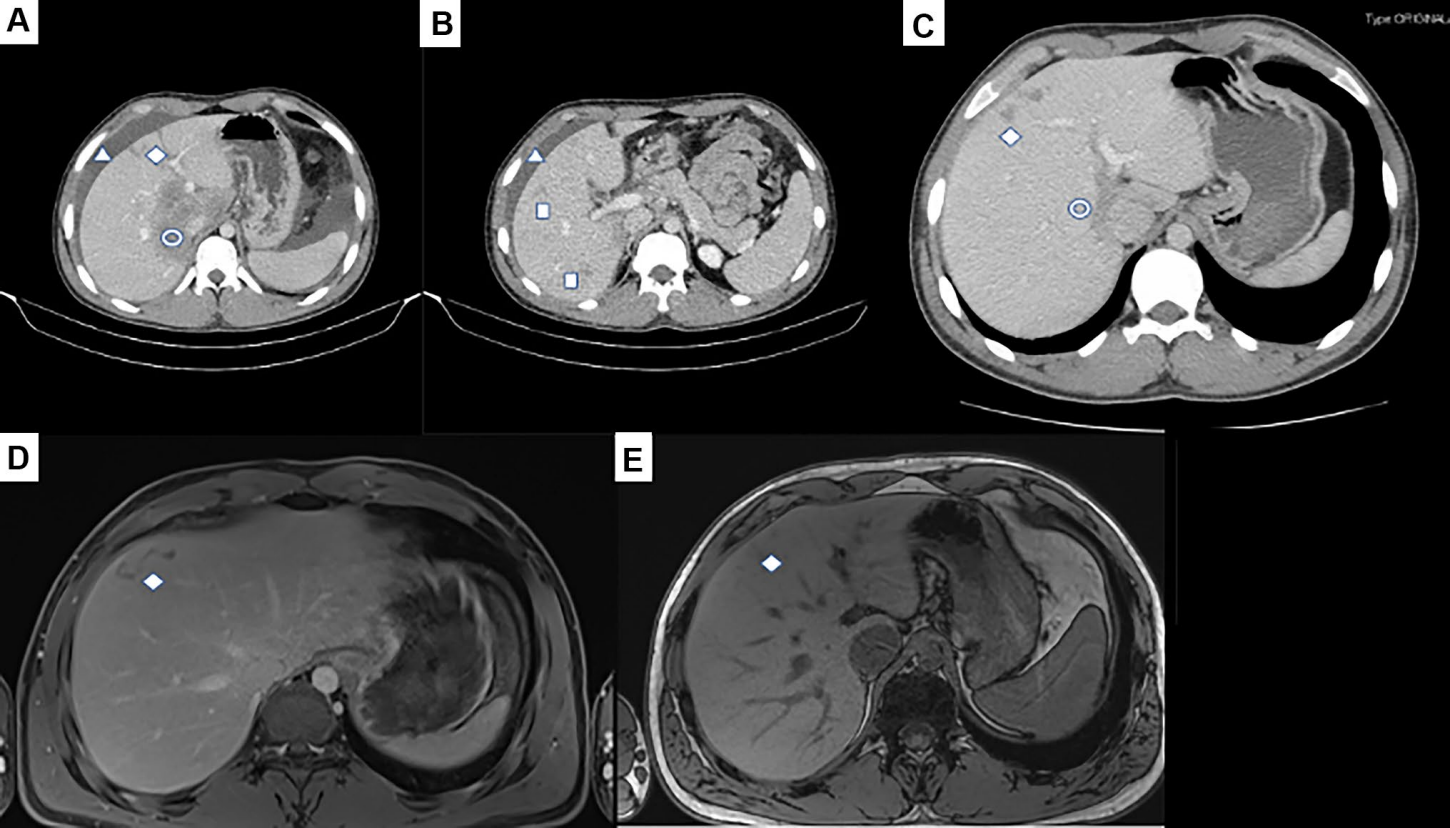
**A** Shows initial CT of the patient as seen at the primary level hospital near his home, August 2021. A hypo-intense peri-caval lesion (segments V, VIII and I, double circle sign) together with a subcapsular lesion in the V segment (rhombus sign). Peri-hepatic effusion is also present (triangle). **B** Shows the smaller, satellite lesions (square signs). **C** Shows the CT scan carried out in November 2021, where a reduced (2 × 2.6 cm vs 7 × 7 cm) porto-caval lesion is evident, whilst satellite lesions were mostly resolved and the subcapsular serpiginous lesion persisted (rhombus sign). **D** Shows an MRI of the liver carried out in January 2022, documenting the persistence of a subcapsular serpiginous lesion in the V hepatic segment (rhombus sign). **E** Documents the near disappearance of the lesion first seen in the V segment of the liver (rhombus sign), documenting radiological improvement

A 19-year-old man presented to our clinic in September 2021. He was healthy and lived in a mountainous region of Tuscany (the Apennines) where he was often in contact with dogs and cats and engaged in outdoor activities (like camping and bathing in rivers). The patient also reported a recent rat infestation in his house. He did not have a history of travel abroad. In late August 2021, he developed high fever (> 38 °C) and abdominal pain that brought him to the ER 8 days from the onset of symptoms. Clinical examination showed no abnormalities of the skin and the patient denied itching. Blood tests showed absolute and relative eosinophilia (12,900 cells/mm^3^, 59.30%), as well as an increase in serum transaminases (AST 147 U/L; ALT 65 U/L) and alkaline phosphatases (227 U/L). The patient underwent an abdominal ultrasound and computer tomography (CT) that showed a hypodense lesion of 70 × 70 mm in the porto-caval segments of the liver, with contrast enhancement in the late portal phase and peritoneal peri-hepatic effusion. Multiple smaller satellite lesions were present and were deemed of infiltrative nature by radiologists. A serpiginous lesion was also present in the V segment. Ascites was also evident (Fig. 1A, B).

He was transferred to a secondary care hospital with an initial diagnosis of liver abscess and empirically treated with piperacillin/tazobactam due to an increased procalcitonin level (3.97 ng/ml) and persistence of high fever. He was also serologically tested for amebiasis and echinococcosis, as well as for leishmaniasis by PCR on peripheral blood all returning negative.

The patient was seen by a haematologist for suspected lymphoma and a biopsy was requested for confirmation given the presence of FHLH.

 He then underwent a hepatic biopsy and was discharged from the hospital due to clinical improvement after antibiotic therapy. He was seen at the Referral Centre for Tropical Diseases of Tuscany in Florence, in September 2021 after biopsy results showed the presence of abscessualizing inflammation and eosinophilic granulomas in the liver. By then, ascites had decreased, but hypoechoic serpiginous lesions with blurred edges were still present in the V segment under the liver capsule at ultrasound (Video 1 - Supplementary materials).

ELISA serology for both *Toxocara* (CHORUS Toxocara IgG, Antwerp, Belgium, index 2.5; cut-off 1) and *Fasciola* (Fasciola IgG ELISA, DRG Instruments GmbH-Germany 1.16; cut-off 1.0) was positive. *Toxocara* serology was also positive by Western Blot (Toxocara Western Blot, LDBIO Diagnostics, France). Given the higher serological title for toxocariasis, the patient underwent an ophthalmological evaluation negative for ocular involvement and was started on a 7-day course of albendazole (ABZ) 400 mg bid, in association with prednisone.

Nine days after the end of the first course, consultation with a toxocara expert (CMC) led to a repeated 2-week course of ABZ and prednisone, given the size of the initial lesion seen on the CT scan (Fig. 1A, B). Treatment with triclabendazole was not started as we considered toxocariasis as the most likely diagnosis, but due to the possibility of the lesion being due to *Fasciola hepatica* instead of *Toxocara*, we obtained several searches for ova and parasites over the course of the following months (21 stool samples over a period of 6 month after the onset of symptoms), all negative for *Fasciola* eggs or other parasites.

In October 2021, a follow-up visit showed significant clinical and laboratory improvement, in particular normalisation of eosinophilia and transaminases, whereas an abdominal CT scan performed in November 2021 showed a reduction of the portocaval lesion and a persistence of the subcapsular lesion, with almost complete resolution of the satellite lesions (Fig. 1C). An MRI of the abdomen performed in January 2022 showed the persistence of the serpiginous lesion in the V segment (Fig. 1D). An MRI in July 2022 showed the permanence of serpiginous lesions in the V liver segment: these were interpreted as inactive (Fig. 1E).

**Fig. 2:**
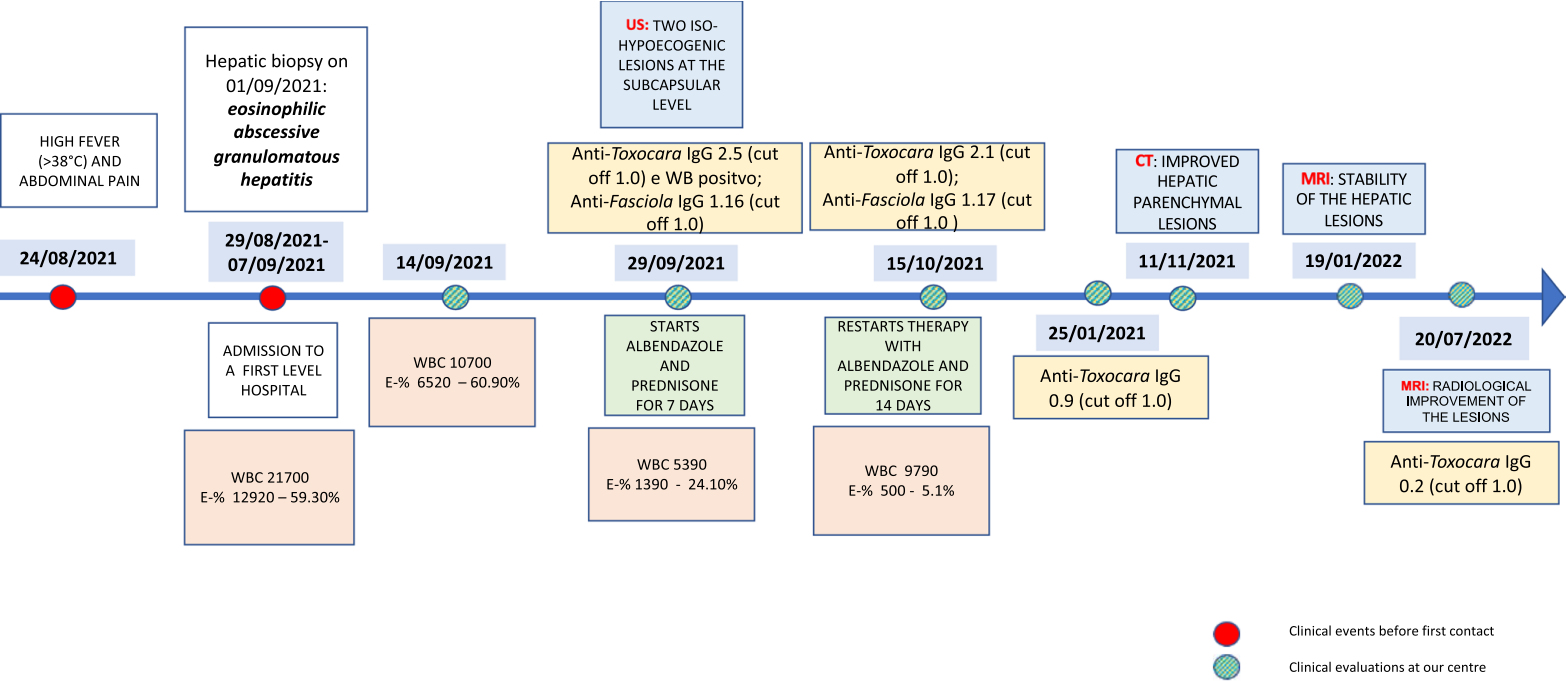
Timeline detailing our case history, including clinical, radiological and serological data for our patient. *WBC* white blood cells, *E* eosinophils, *US* ultrasound, *CT* computer tomography, *MRI* magnetic resonance imaging

As for serological follow-up, we noted a slight decrease in the *Toxocara* IgG titles (index 2.1) and a stable, weak positivity for *Fasciola* IgG (index 1.17) at 1 month from the first serological determination. A further serological sample was collected in January 2022, with a borderline result for *Toxocara* IgG (index 0.9). In July 2022, the patient became seronegative for *Toxocara* IgG. *Fasciola* serology was not repeated due the need for the patient to pay for an exam that was deemed clinically less relevant than before. Triclabendazole was never used given the favourable clinical course and the repeatedly negative ova and parasites searches. Figure 2 shows the chronological order of events in our case report.

## Case Discussion

Toxocariasis has been increasingly reported worldwide, and data suggest it might be a non-negligible problem in Italy: a recent serosurvey conducted in an urban context in Southern Italy found an 8% seroprevalence in the general population [14]. Previous surveys had found lower prevalences (1.6% and 4%, respectively) in northern Italy [7]. The infection is mainly acquired by contact with infected animals (mainly infected puppies) via ingestion of eggs, or by ingesting L3 larvae present in contaminated meat (mostly from wild animals) [10, 11]. Other causes of VLM include less commonly found parasites, such as *Gnathostoma spp* [16] and *Baylisascaris spp* [9]. Capillariasis, caused by *Capillaria hepatica*, is a rarer infection which may have a very high fatality rate and classically present with fever, hepatomegaly and peripheral eosinophilia and is accounted by some authors amongst the cause of VML [12]. Fascioliasis (due to trematodes belonging to the genus *Fasciola*) is also a possible cause of focal liver lesion associated to eosinophilia: the parasitosis is mainly acquired by consuming raw watercress in endemic areas, as well as other vegetables. It is classically believed to be present in South America and the Pacific Region, but experts have suggested that it should be considered a worldwide emerging infection [4]. Fascioliasis usually causes migrant lesions in the liver as the worms actively move from the peritoneum into the liver and subsequently into the biliary tree [11]. Cystic echinococcosis can also be associated with hyper-eosinophilia; however, this usually happens in the presence of complications such as cyst rupture or superinfection [3]. The differential diagnosis of hepatic lesions can be problematic for clinicians working in primary care centres, as the limited number of cases of VLM in Italy lead to these manifestations being confused for other diseases.

Our patient was first issued a diagnosis of suspect lymphoma. This is not uncommon in patients with focal splenic or hepatic lesions. However, risk factors for infectious etiologies should be used to guide differential diagnosis. Our cases (e.g. outdoor activities, close contact with animals that are known vectors for parasitic pathogens, consumption of raw food) [4, 10] were factors that could have suggested a parasitic cause.

In fact, our patient underwent an invasive procedure when the use of a serological exam would have been enough to suggest the presence of a parasitic aetiology.

Our case, however, also illustrates a common issue faced by centres working with parasitic diseases. The positivity of both anti-*Toxocara* IgG and  anti-*Fasciola* IgG led to uncertainty in treatment choice.

The clinical picture was also unclear: whilst fascioliasis is more frequently associated with a peri-hepatic effusion (due to the parasite crossing into the liver from the peritoneum) [4] than toxocariasis, we were torn between the two diagnoses. Since toxocariasis is more frequently reported in our setting and considering the serological results, we decided to treat the patient with ABZ, using a more prolonged course than the 7 days of treatment that are usually recommended [10, 11] after expert advice.

ABZ is also active against the majority of helminths causing VLM. Echinococcosis was also thought of during the patient’s hospitalisation. However, lesions from *Echinococcus granulosus* are cystic in with features defined by a WHO-endorsed classification [1], which were completely absent in the lesions seen in our case. Fascioliasis is included in the foodborne trematodiases present in the list of Neglected Tropical Diseases (NTDs) prioritised by WHO [18], whilst toxocariasis is not.

Recent data have shown that the pathogenetic role of *Toxocara* and *Fasciola* has probably been underestimated in the past [10]. The lack of surveillance programme for these diseases in both animals and humans contributes to our ignorance of the distribution of these pathogens.

When programmes are present, a different issue is the lack of integration of human and animal data and of coordination between surveillance programmes [18]. In fact, this is one of the key aspects to the integrated approach to zoonotic diseases advocated for by the WHO, which is considered a key component of efforts carried out within the WHO roadmap for NTD control ending in 2030 [18].

In our case, a diagnosis of either toxocariasis or fascioliasis was possible according to serological results. Definitive diagnosis of VLM due to *Toxocara* is obtained very rarely through the identification of the larva in bioptic samples, whilst in the majority of cases the diagnosis remain “probable” and based on compatible clinical features and positive serological test [10]. The decrease in size of liver lesions and the lowering title for *Toxocara* after ABZ treatment [10, 11] and the absence of *Fasciola* egg excretion at multiple parasitological stool examinations led us to propend for the former diagnosis. However, we cannot completely role out other helminthic infections associated to VLM (due especially to different less common nematodes), responding to ABZ and which may have cross reacted at the serological test. Since a reactivation of the lesions has been known to occur with parasitic larvae able to escape the granuloma after a dormancy period, the patient will undergo continued monitoring of the lesion [5].

Cross-reactions in serological assays used for parasitic diseases are a known problem, due to several factors including the lack of standardisation in techniques and antigens used for the assays [19].

In conclusion, our case highlights the challenge posed by FHLH and the need to include infectious causes in the differential diagnosis. Amongst parasitic etiological agents, serological cross-reactions can render a definitive diagnosis very hard to reach.

### Supplementary Information

Below is the link to the electronic supplementary material.

Video 1 - Ultrasound of the patient showing two iso-hypoecogenic lesions at the subcapsular level, 29/09/2021. The ultrasound shows a detailed image of the serpiginous lesion seen in Fig. 1 (AVI 3815 kb)

## Data Availability

Data on this case report is available from the authors upon reasonable request.
